# The basic income for care leavers in Wales pilot evaluation: Protocol of a quasi-experimental evaluation

**DOI:** 10.1371/journal.pone.0303837

**Published:** 2024-10-18

**Authors:** David Westlake, Sally Holland, Michael Sanders, Elizabeth Schroeder, Kate E. Pickett, Matthew Johnson, Stavros Petrou, Rod Hick, Louise Roberts, Guillermo Rodriguez-Guzman, Dimitris Vallis, Patrick Fahr, Zoe Bezeczky, Vibhor Mathur, Harriet Lloyd

**Affiliations:** 1 Cardiff University, School of Social Sciences, Cardiff, United Kingdom; 2 Kings College London, The Policy Institute, London, United Kingdom; 3 University of Oxford, Nuffield Department for Primary Care Health Sciences, Oxford, United Kingdom; 4 University of York, Health Sciences, York, United Kingdom; 5 Social Work, Education & Community Wellbeing, Northumbria University, Newcastle, United Kingdom; 6 Centre for Homelessness Impact, London, United Kingdom; University of Bristol, UNITED KINGDOM OF GREAT BRITAIN AND NORTHERN IRELAND

## Abstract

**Background:**

This study will evaluate the Basic Income for Care Leavers in Wales pilot (BIP), which is the most generous basic income scheme in the world. A cohort of care-experienced young people who become aged 18 during a 12-month enrolment period (July 2022-June 2023) are receiving £1,600 (before tax) per month for two years, and the Welsh Government intends this to have a range of benefits. This evaluation will examine the impact of BIP, the implementation of the pilot and how it is experienced, and its value for money.

**Methods:**

The study is a theory-based quasi-experimental evaluation, and the design and methods are informed by ongoing co-production with care-experienced young people. We will estimate the impact of BIP on participants using self-reported survey data and routinely collected administrative data. This will include outcomes across a range of domains, including psychological wellbeing, physical and mental health, financial impact, education, training and volunteering. Comparisons between temporal (Welsh) and geographical (English, using administrative data) controls will be done using coarsened exact matching and difference in differences analysis. The process evaluation will examine how BIP is implemented and experienced, primarily through monitoring data (quantitative) and interview, observational, and focus group data (qualitative). The economic evaluation will take a public sector and a societal perspective to identify, measure and value the costs and outcomes of BIP, and to synthesise the evidence to inform a social cost-benefit analysis at 24 months post-intervention.

**Discussion:**

BIP is unusual in that it targets a wide range of outcomes and is available to an entire national cohort of participants. The evaluation also has several practical constraints. Therefore, the study will use a range of methods and triangulate between different analyses to assess how successful it is. Findings will inform policy in relation to care leavers, social security and basic income studies worldwide.

## Introduction

### Background and rationale

This study will evaluate the Basic Income for Care Leavers in Wales pilot (BIP). The BIP aims to improve outcomes for care experienced young adults across many areas of their lives, and follows other pilots and programmes around the world which have tested the efficacy of basic incomes for disadvantaged groups [1]. The BIP is inspired by research and scholarship on Universal Basic Income (UBI) and is a unique example of a basic income experiment. This is because of both the level of income participants receive and the fact that a whole national age cohort of care leavers is eligible. All care-experienced young people turning 18 during the enrolment period (12 months; July 2022 – June 2023) are eligible to receive a monthly (or twice monthly) unconditional cash transfer from the month after their 18^th^ birthday for 24 months. Participants receive £1,600 gross per month, which is taxed at source leaving a net amount of £1,280 a month. Some participants may be able to claim some tax back depending on their individual circumstances. These figures were based broadly on the Real Living Wage [2] for a full-time employee in 2021/22 (the time of the policy development), and compares to a National Minimum Wage for 18 year olds at the same time of £1,100 per month. This makes this pilot the most generous scheme of its kind worldwide [3]. Similar schemes have targeted homelessness, unemployment, and various other social issues. However, at the time of announcement the BIP was only the second to target care leavers. The first to target this population was a pilot in Santa Clara county, California, USA, which targeted foster care leavers and is discussed below. There are now a series of pilots underway across California with foster care leavers, the definition of which appears to include young people who have been in a range of care settings (not just what UK readers would recognise as ‘foster care’).

#### The Welsh policy and practice context

Wales is a country within the United Kingdom (UK) and has its own devolved administration. The Welsh Government designed the BIP to serve policy goals set out in the Programme for Government 2021-26 and the Wellbeing of Future Generations (Wales) Act (2015), including ‘a healthy Wales’, ‘a more equal Wales’ and ‘a Wales of more cohesive communities’ [4]. In order to design the BIP the Welsh Government set up a governance structure comprising three groups: the Steering Group; the Operations Group; and the Technical Advisory Group (TAG). A range of government officials and experts sit on these groups, with the TAG being chaired by Professor Sir Michael Marmot. BIP also incorporates a human rights approach, particularly the socio-economic duty (Section 1 of the Equality Act) which was commenced in Wales in 2021.

The rationale for targeting care leavers was based on evidence in Wales [5], the UK [6] and internationally [7] that care-experienced young people often face a precarious period in the years after their 18^th^ birthday. A myriad of challenges include a higher likelihood than the general population of experiencing poverty and precarious housing situations [8], and over-representation in the criminal justice system in England and Wales (both as victims and perpetrators) [9]. Early childhood trauma, sometimes compounded by instability in care after becoming looked-after, can lead to a greater propensity for poor mental and physical health [10]. Notwithstanding these wide-ranging challenges, many care-experienced young people achieve successes in education, employment and contribution to their communities [11].

Risks to children tend to be greatest before and after they are in care, and there is evidence that the benefits many children gain from being in care risk being undone in the years after they leave [12]. The outcomes for children in public care are generally considered to be poor. This has contributed to a focus on reducing the number of children in care: a goal that is made explicit in the provisions of the current Children and Young Persons Bill. Yet while children in care do less well than most children on a range of measures, such comparisons do not disentangle the extent to which these difficulties pre-dated care and the specific impact of care on child welfare. Moreover, the service offer for care leavers in Wales has improved in recent years, with more options and support for young people reaching adulthood. It is encouraging to note that the most common housing destination for care leavers at 18 is now to stay with their foster carers in a ‘When I’m Ready’ arrangement [13]. Other welcome advances to tackle poverty amongst care leavers in Wales include exemption from paying Council Tax [14] and the St David’s Fund, which is administered by local authorities and designed to support young people who are or have been in care to gain independence [15]. These initiatives are additional to longer-standing financial support programmes such as Higher Education bursaries and cost of living payments. Alongside this progress, BIP will be seen by many as a step change in the Welsh Government’s efforts to alleviate poverty, and its associated negative impact, amongst this group.

#### Overview of basic income schemes

The concept of a basic income (BI) can be traced back to ancient Greece, where Pericles (461 BC) was thought to have instigated a payment to citizens that resembled a basic income [16]. The idea has been developed and a modern definition of universal basic income offered by Van Parijs suggests it is “unconditionally paid to every member of a society [1] on an individual basis [2] without means testing and [3] without work requirement” [17]. The 1970s witnessed a few Negative Income Tax experiments in the USA, and the first pilots close to a basic income are considered to have taken place in Manitoba and Dauphin in Canada [18]. In the 21^st^ century there has been a sharp increase in the number of basic income trials around the world, including a few which are large-scale and a few that are government backed [19]. They are seen to serve two main purposes; to demonstrate feasibility and to evaluate effects [19,20]. The terms ‘pilot’, ‘experiment’ and ‘trial’ are often used interchangeably, though some have used these terms to distinguish examples of BI according to how far they adhere to theoretical ‘ideals’. Torry (2023), for example, argues only examples funded by tax revenue and with a meaningfully representative group and should be called ‘pilots’, even though this is often impractical and here are few examples of schemes that are truly universal [21]. There are however a growing number of pilot schemes aiming for community saturation, and where recipients of BI typically receive an income for which eligibility is not means-tested or dependent on (searching for) employment. (We use ‘pilot’ in the original sense of the term, and not only to mean an ‘ideal type’ scheme as suggested by Torry (2023)).

Pilot schemes vary according to their universality, conditionality, regularity, duration, how they interact with existing provision, the amounts of money offered, and how they are funded. Basic income schemes and similar programmes encompass a somewhat disparate range of arrangements, such as UBI, targeted cash transfers, social dividends, guaranteed annual income, guaranteed minimum income and negative income tax [1]. Care leavers in the BIP receive monthly or twice-monthly payments larger than that of any previous trial. Several other pilots of BI schemes have been undertaken across the world in recent years, and schemes are currently underway in numerous countries, including over 55 in the US alone [22].

A high-profile Finnish trial was delivered over the whole of 2017 and 2018 [23]. Participants were 2,000 people aged 25-58, from across Finland, who received unemployment benefits. They were selected at random to receive a tax-free payment of €560 per month, for the two-year duration of the trial. This was equivalent to, and in place of, the net level of basic unemployment benefit and basic income recipients remained eligible for other benefits (e.g., housing allowance or social assistance [24]). Participation in the trial was mandatory for the intervention group, and 178,000 unemployed individuals formed the control group [24]. The findings of the Finnish pilot have been widely debated, and are complicated by complementary interventions making attribution difficult [25]. However, the more recent and comprehensive analyses have shown that the number of days in paid employment were moderately higher for the basic income group rather than the control group, even if this cannot be fully attributed to the basic income. Additionally, the basic income group reported much higher levels of wellbeing, fewer physical and mental health issues, and higher life satisfaction than the control group [25,26].

Several of the ongoing schemes are particularly relevant to the BIP, in terms of their aims and target population. In the US, the California Department of Social Services are funding seven pilots across the state that target young people leaving foster care at or after 21 [27]. Almost 2,000 individuals will receive $600 to $1,200 monthly payments for between 12 to 18 months, depending on the pilot. The charity iFoster is running the biggest pilot for young people who are aging out of foster care [28]. They are providing 300 young people with $750 per month for 18 months. The pilots are due to end in 2025, and they are being evaluated by the Urban Institute, Washington DC, and the University of California, Berkeley [29–31].

#### Research on basic income

Evidence on the impact of BI is promising but incomplete. Encouraging evidence has emerged from several recent reviews which have assessed the evidence base on the effectiveness of basic income and similar schemes [1,32,33]. These reviews show that studies have predominantly assessed impact on health, education and employment outcomes.

Gibson et al’s (2018) study was a scoping review of schemes that resemble BI, such as those associated with resource extraction dividends in Alaska, casino dividend payments for Indigenous Americans, and negative income tax schemes for low-income families. They show consistent positive impacts on health, education, entrepreneurship and crime. Specific health benefits reported include a greater uptake of health services and improved food security, nutrition, birthweight, and adult and child mental health. There is also evidence that the schemes positively impact education outcomes, with more consistent evidence for short-term (e.g. school enrolment and attendance) rather than long-term outcomes (e.g. attainment). Several studies have reported improved family relationships, more suitable housing arrangements, and reductions in adolescent and adult criminal behaviour [34–37]. However, there have also been some examples of adverse outcomes, such as increased substance misuse among individuals who took part [38,39].

Individual-level outcomes such as these have been the focus of most studies to date, and consequently outcomes at the community level have not been studied extensively. Nonetheless, there have been reports of “spill over” effects when payments are made to a large proportion of a population [40,41]. These include an increase in business activity and reduction in hospital admissions [18,42,43]. Positive effects were especially notable where payments were sufficient to meet basic needs and were made regularly, rather than as annual lump sums [44].

There is also evidence that the schemes have a minimal impact on labour market participation, which is important because some criticism of basic income schemes is based on the assumption that a basic income reduces labour market participation by removing incentives to work [45]. For the Finnish trial, Verho et al. [46] studied employment effects through administrative data and found no statistically significant effect on days in employment in the first year of the experiment. They found that employment effects were somewhat higher in the second year of the experiment with an increase in employment days among the intervention group (6.6 days (95% CI 1.3-11.9; p = 0.01)) [47]. Several studies utilising survey data in Finland, with a relatively low average response rate of 23%, have similarly found no significant effects on employment but greater confidence among the intervention group about the future, their ability to cope with difficult life situations, possibilities to improve their economic situations and find employment and higher levels of trust in the social security system [47–49].

**Fig 1:**
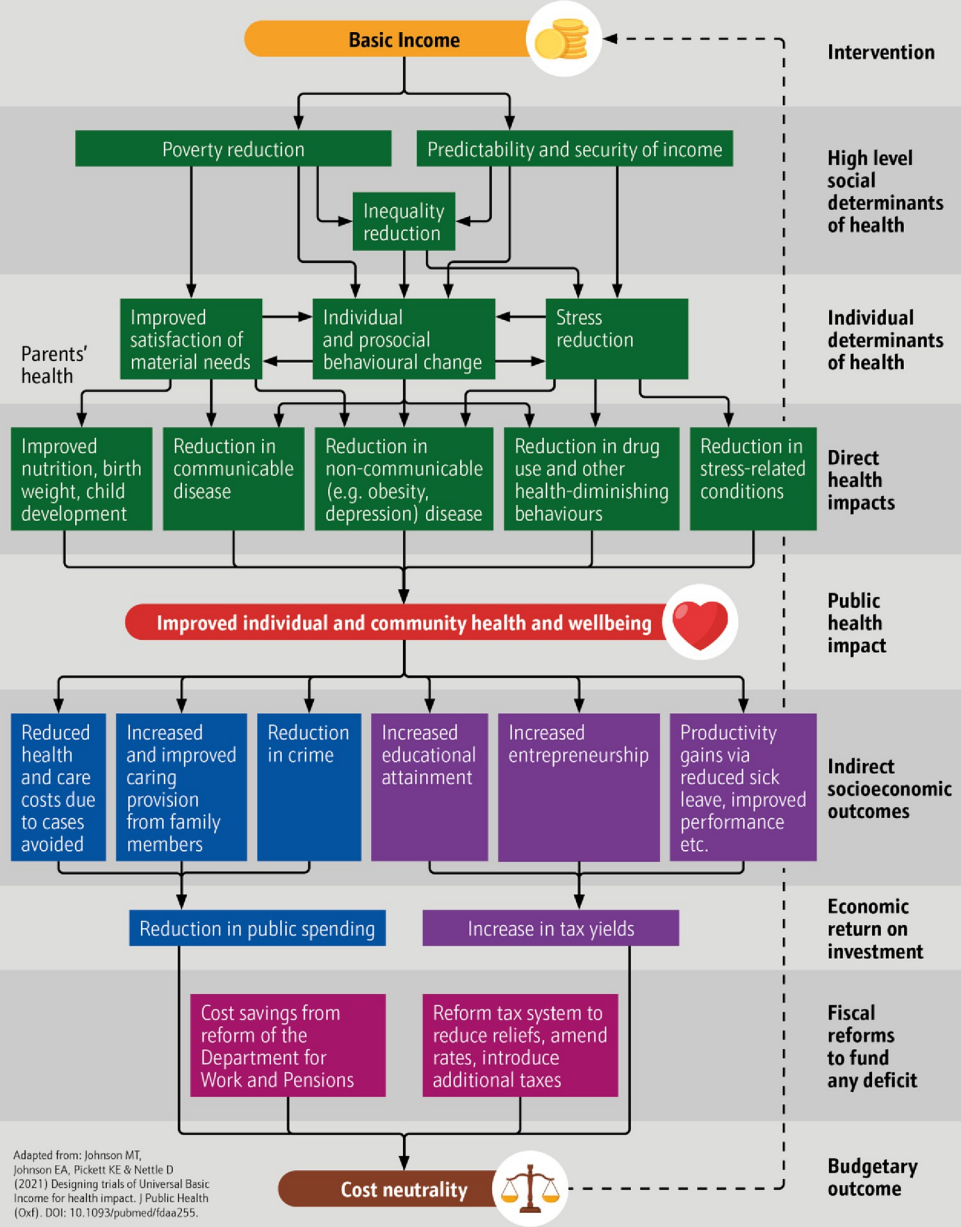
UBI model of impact (adapted from Johnson et al, 2021 [51]).

However, the existing evidence base gives an incomplete picture because none of those schemes have been evaluated using a comprehensive range of outcomes or with methods that allow impact to be compared across studies [50]. Johnson, Johnson, Pickett and colleagues [51] suggest that impact on individual educational, economic, social and health outcomes (and attendant impact on public budgets) is likely to be significant. Their logic model (Fig 1) suggests that where UBI increases the size of income, it can reduce poverty [52]; when it increases the security of income, it can reduce stress associated with threat of destitution [53]; and when it makes income more predictable, it can improve the social determinants of health, promoting longer-term thinking and behaviour that improves outcomes [51]. They have set out a generic, adaptive protocol resource to measure these impacts in basic income trials and this has informed our design [44].

The highly politicised nature of basic income experiments underlines the importance of clear and comprehensive evaluation. The scheme in Ontario, that was abruptly cancelled after a change in government, illustrates the potentially negative effects of political intervention. This resulted in no evaluation findings due to a lack of data and the fact researchers are bound by confidentiality agreements [54]. Even in cases where evaluations have reported as planned, an absence of comprehensive evaluation or clarity around anticipated outcomes has sometimes left pilots and trials of basic incomes vulnerable to ‘spin’ [21,55]. This is partly related to how findings have been framed in the published literature. As noted above, the Finnish trial found no negative impact on labour market participation. However, evaluators failed to make clear that the expectation of critics of basic income was that it would cause a *reduction* in labour market participation through ‘free-riding’, and hence that no change represented a finding in favour of BI. For example, Verho et al [46] concluded “The Finnish experiment failed to produce any sizeable short-term employment effects despite offering larger improvements in employment incentives than any realistic nationwide policy could provide” (p. 27), without acknowledging this context.

A more fundamental weakness of that trial is that it failed to measure health impacts comprehensively using validated measures in ways that would have advanced evidence on the pathways and nature of causality. Similarly, the Finnish trial presented positive findings in relation to subjective wellbeing, but without baseline data, which meant these could not be attributed to BI [25]. The absence of robust and validated outcome measures that could also be used by health economists deprived the trial of key evidence on overall costs and benefits, with recent work by Johnson, Johnson, Pickett and colleagues highlighting the importance of employing comparable measures that can be used in microsimulation to model longer-term impacts at population level [44]. A weakness of the evidence base more generally is that long-term impacts have not been adequately evaluated due to the relatively short duration of most pilots [56].

#### The basic income for care leavers in Wales Pilot

The level of income participants receive in the BIP makes it unique among basic income pilots, but other aspects of the scheme are also notable. In targeting care leavers, it bears similarity to the Californian pilots mentioned above. Yet, unlike these pilots, which are focussed on foster care leavers, the BIP includes young people who have been in other placement types, such as residential and kinship care. (Residential care in the UK is a form of group care for looked after children, where care is provided by teams of paid staff. Kinship care in the UK is where a looked after child resides with members of a relative, friend or other connected person – usually a member of their extended family.) Although evaluation results are not yet available from the Californian pilots, it is clear they differ in several other ways from the BIP. The Santa Clara County pilot offers older foster care leavers a lower amount for less time. The 24-year-olds involved receive $1,000 (circa £785) per month for 18 months (after the initial 12-month period was extended). It also includes fewer participants which may make it more difficult for evaluators to estimate effects robustly, though a pooled analysis of the Californian pilots may overcome this.

The aims of the BIP in Wales are also somewhat unique and demand a broad-based assessment of impact that includes a range of types of outcomes. In explaining the rationale for the Basic Income for Care Leavers in Wales pilot, the Welsh Government cites empowerment as a key aim: they hope the pilot will help care leavers feel more able and confident to make decisions, navigate challenges, and engage with their communities. Some of the more specific outcomes the Welsh Government are targeting overlap somewhat with the Californian pilots aims around poverty, equity and basic needs, but they also extend to other types of outcomes. As some of these aims are less amenable to quantification than other outcomes, it is important for the evaluation to assess these qualitatively. In a statement outlining the pilot, the Welsh Government Minister for Social Justice set out four key principles for the scheme [57]:

1. Taking part in the pilot should make no participant worse off
2. There should be no conditionality on income received
3. The same payment should be paid to everyone
4. The payment will not be altered midway through the pilot.

Unlike some other basic income pilots, most notably the Finnish trial, the BIP in Wales was not set up as a research study. The evaluation is therefore designed around an existing policy, which has various implications that we discuss herein.

### The current study

#### Objectives

This study is designed around three linked objectives. The primary objective is to evaluate the BIP in terms of its impact, how it is implemented and experienced, and its value for money. These are the three core areas of analysis that we will report on. The second and third objectives, respectively, are to contribute to the international evidence bases summarised above, around basic income schemes and around support for care leavers.

#### Research questions

The research questions (RQs) listed below relate to the three core areas of (1) impact evaluation, (2) implementation and process evaluation (IPE), and (3) economic evaluation. Within the IPE we will explore implementation, experiences, and integration with existing services. Sets of sub-questions are emerging through discussions with our co-production group and early IPE data collection.

- RQ1: What is the impact of BIP?
- RQ2: Is the pilot implemented as intended?
- RQ3: How is the pilot experienced?
- RQ4: How does BIP fit into the overall offer for care leavers in Wales?
- RQ5: How cost effective is BIP?

#### Study setting

The study will be conducted in Wales across all 22 local authority areas. Local authorities are the lowest level of elected government in Wales, and are responsible for delivering Children’s Social Care Services.

#### Delivery and evaluation structure

Several parties are involved in delivery of the intervention and the evaluation. The Welsh Government is delivering the intervention in partnership with the 22 Welsh local authorities. The Welsh Government has also commissioned NEC Software Solutions UK to administer the payments. The advocacy group Voices from Care Cymru provide advice and enable co-production. Citizens Advice provide support and advice to eligible care leavers. The evaluation is being delivered by our consortium of Cardiff University (lead), King’s College London, University of Oxford, University of York, Northumbria University and the Centre for Homelessness Impact. Coram Voice, a children’s rights charity, and University of Oxford’s Rees Centre, have been separately commissioned by WG to deliver surveys (details below).

#### Intervention and comparator conditions

The intervention group receive a basic income once or twice a month for two years, once they join the pilot in the month following their 18^th^ birthday. Those opting to receive the transfer twice monthly will receive two payments of £800 gross (£640 net); those receiving a monthly transfer receive one payment of £1,600 gross (£1,280 net). The amount of money received as a basic income was calculated by the Welsh Government to be broadly similar to the ‘Real Living Wage’ for a full-time employee in 2021/22, and it equates to £19,200 (gross) annually. This income is treated as unearned income for tax and benefit purposes and taxed at source at the basic rate of tax (20%), meaning that enrolment on the scheme may change participants’ entitlement to other benefits or liability for taxes (e.g. income tax). Individuals in the intervention group are also eligible to receive advice from the Citizens Advice Cymru, provided through the Single Advice Fund including a ‘better off’ calculation to determine whether enrolling on the pilot is financially beneficial. Comparisons will be made between the intervention group and comparator groups, including care leavers in Wales with 18^th^ birthdays in the 12 months following the enrolment period, and care leavers in England who are the same age as the intervention group. The comparator groups will not receive the basic income but will remain eligible for other benefits depending on their circumstances.

#### Strategies to improve adherence to intervention

Take-up of the intervention is high, and 97% of those eligible for the scheme enrolled onto it [58]. The Welsh Government has worked with local authorities to support take-up and the strategies used will form part of the implementation analysis. However, the intervention is voluntary and some eligible individuals may choose not to participate. A ‘better off’ calculation is carried out for each care leaver at the point of enrollment, to ascertain whether enrolling is in their best interests financially. The IPE is designed to capture these data and will explore the reasons and consequences of not participating.

#### Eligibility criteria

All young people who are ‘Category 3’ care leavers turning 18 years of age between 01 July 2022 and 30 June 2023 are eligible. Category 3 care leavers are those who are aged 18 or over who spent at least 13 weeks in the care of the local authority after the age of 14 and were still in care on their 16^th^ birthday (Social Services and Wellbeing (Wales) Act 2014, S.104). Children in local authority care may be looked after in foster care, residential care, kinship care or be placed with their parents under a Care Order in which parental responsibility is shared between the legal parents and the local authority.

## Methodology

The study design is an impact evaluation based on a quasi-experimental design (QED), with integrated implementation and process (IPE) and economic (EE) evaluations. These are situated within a theory-based approach and a commitment to co-production, which will guide all aspects of the study. Theory-based approaches [59] are optimal for evaluating complex social interventions. We are aiming to understand whether BIP changes specific outcomes, how it may have these effects, why and for whom it may be beneficial or detrimental, and under what conditions these changes may happen [60]. Logic models that delineate the mechanisms that underpin the anticipated effects of BIP, and of basic income schemes more broadly, provide a basis for ‘theory enhancement’ using data from this evaluation. An updated programme theory and logic model will therefore be a key output.

Co-production is increasingly recognised as essential to high quality research and policy practice and is in keeping with Welsh legislation and policy, including the Social Services and Well-being Act 2014. Co-production will underpin the study and our participatory methods encompass the entire research cycle, and follow the UK Standards for Public Involvement in Research (NIHR) [61] and Wales’s Participation Standards [62]. A group of care-experienced young adults, living in a range of educational, employment and housing situations, will meet for a minimum of 16 sessions to advise the research team. Their role is to co-create research questions, data collection instruments, consider ethical and analytical questions and advise on policy and practice implications.

### Impact evaluation

To measure impact the design incorporates a suite of quasi-experimental designs (QEDs) which will enable triangulation between multiple data sources and provide a robust account of the difference the BIP makes to care leavers in Wales. QEDs attempt, in the absence of randomisation, to achieve identification of the causal impact of one or more interventions, primarily through a mix of sample selection and statistical approaches [63]. Randomisation is not possible in this case because the BIP is open to all eligible young people and starts at the same point for all participants (the month after their 18^th^ birthday). The QED approach will enable us to determine that, conditional on our sample selection and analytical strategy, we do not expect to see any uncontrolled-for differences between the intervention and counterfactual groups, and so any differences between the two groups can be attributed to the BIP. Further detail about our analysis plan is available in supplementary materials.

We aim to compare the outcomes of care leavers who turn 18 during the enrolment year (and are thereby eligible for the BIP), to outcomes of care leavers in Wales who turn 18 the following year. This will involve two quasi-experimental approaches: coarsened exact Matching (CEM) and difference in differences (DID). Measurement will take place at two time points. Baseline data will be gathered around the individual’s 18^th^ birthday (referred to below as time t-1) and follow-up data will be gathered around their 20^th^ birthday (referred to below as time t).

#### Outcomes and data sources

The Welsh Government identified six outcome domains of interest, and the literature suggests that it is also important to include physical and mental health outcomes more broadly. These, and the means of collection through surveys and administrative records, described below, are outlined in Table 1.

**Table 1:** Outcomes and data sources.

Outcome domain	Collected through	Specific measures
Wellbeing/ psychological Wellbeing	Survey Data	Categorical indicators of frequency of emotional states, level of anxiety, happiness, feelings of positivity, including an open-ended question
Financial literacy/ security	Survey Data	Questions on levels of financial coping, allocation of income, spending habits with an open-ended question on money management.
Community cohesion/ engagement	Survey Data	Binary indicators of friendship, partnership, owning a pet, having a person of trust, and categorical indicators of emotional support. Binary indicators of community cohesion in follow up (t) survey
Ameliorating the effects of poverty	Survey and administrative data	Categorical indicator of current housing, binary indicator of housing satisfaction Leisure and access to luxury items/internet
Access to labour market/ education/ lifelong learning	Administrative data	Binary indicator of employment; continuous measure of earnings; binary indicator of enrollment in education or training; binary indicator of participation in higher education; binary indicator of participation in further education. These outcomes will be extended using linked longer-term data from the Longitudinal Education Outcomes (LEO) data.
Volunteering and life skills	Survey Data	Education, employment and training.
Physical and mental health	Survey and administrative data	Self-rated general health (ONS question) Limiting long-term illness (ONS question) Common mental disorders: depression (PHQ-2); anxiety (GAD-2).

The Welsh Government have not specified what effect they expect BIP to have on these outcomes, though they aim for the policy to empower participants, give them more agency and control, and improve their lives. The literature on basic income suggests that we should not see any detrimental effects in any of these areas, and that in many areas improvements would be hypothesised. Some of these improvements may take longer than others to materialise, meaning that some benefits may not be detectable during the timescale of the study. This study will publish analysis of the observed effects on all outcomes in Table 1. The measures cited were selected by the evaluation team and approved by the Welsh Government.

*Survey data*. We are contracted to use data already collected by Coram Voice, who are a third-party commissioned earlier by the funder to gather survey data from participants of the BIP. Surveys will be administered by Coram Voice for the intervention group and comparator group at two time points (t-1; around the participants 18^th^ birthday, t; around the participants 20^th^ birthday). It should be noted that both of these time periods differ for each participant in the study, because they turn 18 at different points during the enrolment window. Therefore data collection will, in practice, take place continuously over the study period.

The survey is based on a similar survey used with care leavers extensively in England, called ‘Your Life Beyond Care’ [6]. Coram Voice started collecting data in Wales in October 2022, 4 months after the pilot had started, and initially included only the original questions used in the ‘Your Life Beyond Care’ survey. In January 2023 an updated survey was released with additional questions designed to cover the broader range of outcomes in Table 1. The new questions added were the result of negotiations between the evaluation team, the funder, and Coram Voice. At the point we were commissioned, in November 2022, survey response rates for the original survey were 6%. After several changes to the mechanism for collecting survey data were agreed, and questions added, response rates increased and the final response rate is 64%. The Welsh Government and Coram Voice take informed consent for survey data.

*Administrative data*. We will use the Longitudinal Educational Outcomes (LEO) dataset which is held by the Office for National Statistics (ONS) and the Welsh Government. This resourcelinks educational data from the National Pupil Database (NPD) for England, the Pupil Level Annual School Census (PLASC), the post-16 pupil collection and the Lifelong Learning Wales Record for Wales, employment and earnings data from HM Revenue and Customs (HMRC) and the Department for Work and Pensions (DWP), progression and success in further education from the Individualised Learner Record (DfE) and progression to higher education from the Higher Education Statistics Agency. LEO also contains markers for young people’s social care experience, which will allow us to identify care leavers in both England and Wales. We will initially consider the universe of data on care leavers from England and Wales, and will use a two stage matching process (detailed below and in more detail in our Statistical Analysis Plan), to select a group of English care leavers who are (a) in English local authorities that are comparable to the 22 Welsh local authorities and (b) who are comparable to the Welsh care leavers within those local authorities.

Use of administrative data will allow us to link the intervention group with a large enough comparable group of young people experienced in care during the same period in which the intervention took place. This will provide the analysis with higher power, increasing precision while allowing for a broader range of outcomes to be explored. Administrative data will also allow for a contemporaneous comparison of outcomes between the control and the intervention. This will enhance causal inference with respect to unobserved time-dependent covariates that may have been correlated with the outcome(s) of interest at the time of the intervention, an aspect of analysis which is not possible in the survey-based analysis.

Outcomes within administrative data will be aligned with various outcomes already explored within the survey (see Table 1), thus allowing for accurate inference on the effect of the intervention. For outcomes not covered by the survey, a binary interaction indicator of whether the participant was in a local authority that would make them eligible for the basic income payments at the time of the intervention will be used to capture the treatment effect.

Other administrative data will also be available from the Welsh Government. They are managing the enrolment of eligible young people in collaboration with the 22 local authorities. Each participant completes an enrolment form at the outset, and this includes a tick box for consent to be included in the evaluation. Enrolment forms contain a range of monitoring data, including some self-reported data about the individual’s health circumstances. The Welsh Government are designing an exit process that may mirror aspects of enrolment, but are yet to finalise this at the time of writing. Some data items gathered at the enrolment stage for participants in the intervention group will be added to surveys completed by participants in the comparison group (as these data would otherwise be missing for this group). The Welsh Government will take informed consent for monitoring data to be shared with the evaluation team.

*Sample size*. In the evaluation specification, the Welsh Government advised that around 550 young people were expected to become eligible for the intervention during the enrolment period. (The actual update for the scheme is 635 recipients.) Power calculations are difficult to usefully conduct ex ante for a matched difference-in-differences approach, reflecting the relative complexity compared with the canonical RCT approach. Nonetheless, we anticipate being able to detect effects on survey outcomes of no more than 0.2 standard deviations (calculated via Glass’s Delta), and for effects of no more than 0.12 standard deviations for the administrative data, based on our experience with other similar projects. These effect sizes are comfortably within the range of small effects, allowing us to build a clear picture of the impacts of basic income. However, it should be noted that the small sample size makes subgroup analysis, particularly for any group which is in a minority among eligible participants, difficult to conduct reliably.

*Matching procedure*. Coarsened Exact Matching (CEM) [64] lies somewhere between the two extreme forms of matching - the completely uncoarsened Exact Matching, or the logical extreme of coarsening to a single figure - the Propensity Score [65]. In CEM, matching variables are preserved, but are coarsened. Here, we can think of coarsening as redefining variables into ranges. For example, instead of a participants’ height being an exact number of centimetres, which might be difficult to match in small samples, this could be coarsened to heights in ten centimetres intervals. By doing this, participants continue to be matched on the values of their observable characteristics, but the likelihood of matching on any variable or set of variables is increased.

This matching approach has the advantage of yielding more matches than exact matching, while also ensuring that units are matched on measures that are relevant to the *outcomes* of interest.

For the administrative data, we will use Coarsened Exact Matching at two levels - first to match Welsh local authorities with their English counterparts, effectively matching treated local authorities to statistically *similar* untreated authorities, and second to match care leavers within those local authorities with each other. To identify counterfactual local authorities in England we accessed publicly available information on the age, legal basis, gender, and numbers of care leavers in local authorities in England and Wales from 2018-2022, and merged these into a single dataset with a panel at local authority/year level. This panel was then used to identify variables capturing the rate of change in the children in care within each local authority in England and Wales.

We further accessed data on indices of multiple deprivation for England and Wales, particularly focusing on the indices employment, income and childhood deprivation at MSOA level, which was subsequently collapsed to give a local authority level average for each score for all top tier local authorities in England and Wales (which deliver children’s services). This was in turn matched into the panel dataset created previously.

When matching using Income Scores, IDACI score and IDAOPI score, we identified 41 matches for 21 Welsh local authorities. Using Employment Score instead of IDAOPI score produced 29 matches for 18 Welsh Authorities. Reducing this to only Income scores and IDACI Scores yielded 71 matches for all 22 Welsh local authorities. Given the need for some specificity of matches (more than half the local authorities available for matching are matched in the second model) the first or second approach, which only identifies matches for 21/18 Welsh local authorities are preferred at this stage.

For both of our potential models, we test balance on trends in care numbers, and any omitted scores. We find that our second model, which includes Employment rather than IDAOPI, creates a more balanced sample overall, except for with respect to IDAOPI, which is significantly imbalanced.

For survey data, we will match at care leaver level, matching care leavers in the treatment cohort with statistical neighbours in the subsequent cohort (both cohorts being in Wales). Where possible this will involve matching participants on a range of variables *including* their local authority. Matching for survey data can therefore not take place until after data is collected.

**Fig 2:**
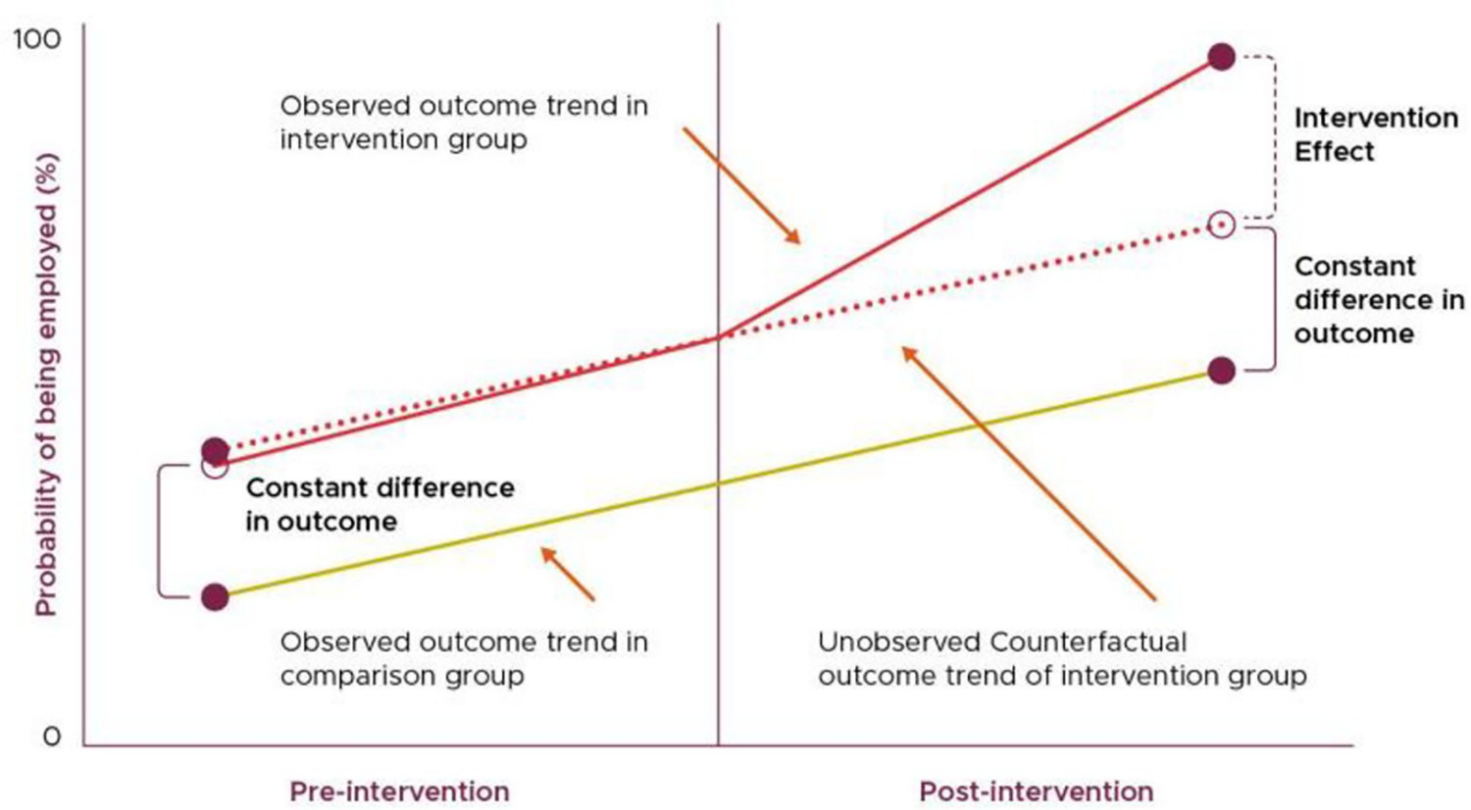
Difference in differences example (reproduced from Sanders and Murphy, forthcoming [67]).

*Difference in differences*. Difference in Differences approaches are quasi-experimental approaches which compare the differences between treated and counterfactual units, before and after the introduction of a new policy or intervention (Fig 1 below). This comparison allows for time invariant differences, whether observed or unobserved to be controlled for analytically [66]. We will undertake two different versions of the difference in difference approach for our two separate data sources. For administrative data, we will make use of a standard difference in difference in which participants in Wales are compared with matched English care leavers, covering the same time period (and same life stage) as the Welsh Data. For our survey outcomes, due to the challenges associated with collecting data from English care leavers, we will instead draw our counterfactual group from the subsequent cohort of Welsh care leavers. Although the comparison of two groups that have not been measured contemporaneously (as is the case comparing Welsh Care leavers in one cohort with Welsh Care leavers in another) is a non-standard implementation of the difference in differences methodology, the underlying assumptions remain the same. Instead of assuming common trends in the macroeconomic conditions (as would be the case for a standard DID), we are instead assuming common trends over the same period of the life course – that is, we assume that outcomes change in the same ways for young people between their 18^th^ and 20^th^ birthday, for young people whose 18^th^ birthdays were up to a year apart. The broad approach to a Difference in Differences approach can be seen in Fig 2.

#### Statistical methods for analysis of impact

Our analytical strategy for the survey data will follow a matched difference in differences approach. Matching will take place prior to analysis in order to select the most appropriate sample.

The difference in difference strategy for this data will be to take the first time period as baseline survey data for both the treatment and counterfactual groups, and to make use of the endline survey data as the second time period.

*Imputation strategy*. Inspection of the missing data pattern will provide some initial insight in the type of missingness, and statistical testing will further help assess whether the missing data mechanism is Missing Completely At Random (MCAR) or Missing At Random (MAR). We will utilise Little’s MCAR test [68], which determines whether the missingness is related to the observed and unobserved data. We will also use a logistic regression model with an indicator of missingness as the outcome, which will show whether relevant covariates are predictive of missingness, pointing towards the plausibility of a MAR assumption.

Due to the nature of survey data collection, we anticipate missing data for some participants. We will not make use of imputation for outcome measures as this carries substantial risks in terms of bias. For missing data at baseline we will make use of a mixture of Multiple Imputation through Chained Equations (MICE) [69], in which available baseline or demographic data for the participant are used in regression analyses to calculate the likely values of the missing variable. Following imputation of missing data, we will examine the convergence diagnostics to ensure that the imputation process is stable and the imputed values are plausible.

*Analytical specification*. *Primary analysis – surveys*. Our primary analysis specification will be conducted using ordinary least squares/linear prediction model regressions, specified as;

$$Y_{ilt}=\alpha +\;\beta_{1}Y_{ilt-1}+\beta_{2\;}B_{l}+\Gamma_{1}X_{i}+\Gamma_{2}L_{l}+\epsilon_{lt}$$

Where;

*Y*_*ilt*_ is the outcome measure for individual i in local authority l at endline.

*α* is a regression constant

*Y*_*ilt*−1_ is the lagged value of the participant’s outcome measure at baseline.

*B*_*l*_ is a binary indicator of whether the participant was eligible for the basic income payments, set to 1 if they are and 0 else (equivalent to a binary indicator for being in the eligible cohort

*X*_*i*_ is a vector of participant level characteristics.

*L*_*l*_ is a vector of local authority fixed effects

*ϵ*_*lt*_ is an error term clustered at the level of the local authority/time period level.

*Follow-up (t) only data*. Some variables in the survey are only collected at follow-up (time t), and not at baseline (time t-1). For these variables, we will adopt a less typical approach and replace *Y*_*ilt*−1_ with a vector of baseline variables that are the strongest control group predictors of the outcome at time t.

*Primary analysis - administrative data*. Our primary analysis specification will be conducted using ordinary least squares/linear prediction model regressions, specified as;

$$Y_{ilt}=\alpha +\;\beta_{1}T_{t}+\beta_{2\;}B_{l}+\beta_{3}(T_{t}\cdot B_{l})+\Gamma_{1}X_{i}+\Gamma_{2}Q_{l}+\epsilon_{lt}$$

Where;

*Y*_*ilt*_ is the outcome measure for individual i in local authority l in time t.

*α* is a regression constant

*T*_*t*_ is a binary indicator of whether or not the time period in question is that in which the intervention is active.

*B*_*l*_ is a binary indicator of whether the participant was in a local authority that would make them eligible for the basic income payments, set to 1 if they are and 0 else (equivalent to a binary indicator for being in Wales).

(*T*_*t*_∙*B*_*l*_) is an interaction term between treatment local authority and treatment time period, which takes values of 1 only for Welsh local authorities during the time period when the intervention is active. This is our treatment variable and its coefficient is our coefficient of interest.

X_i_ is a vector of participant level characteristics.

*Q*_*l*_ is a vector of local authority level characteristics including those used in matching.

*ϵ*_*lt*_ is an error term clustered at the level of the local authority/time period (the level at which the treatment status varies).

*Secondary Analysis*. Secondary analysis will follow the same regression specification as our primary analysis, but replacing the variable Y with the relevant secondary outcomes.

*Robustness Checks*. We will robustness check our analyses by:

- Using Null imputation across the board, replacing MICE
- Conducting complete case analysis
- Using logistic and probit regression for binary outcomes

### Implementation and process evaluation (IPE)

To understand how and why BIP works, for whom and under which circumstances, we intend to use qualitative and quantitative methods to explore research questions in three main areas of enquiry: (1) implementation, (2) experiences, and (3) integration. This strand of the project will be in dialogue with ongoing theory enhancement, in that questions asked will draw on the initial programme theory, and empirical findings will feed back into ongoing theory enhancement.

Table 2 details the objective and research question attached to each area of interest, and how these are served by the data collection activities specified below. Most qualitative data collection will take place with the same participants early in the pilot intervention and repeated as the pilot draws to a close.

**Table 2:** Data collection strategy by research area/ question.

Area/ Question	Objective	Main data sources
Implementation: Is the pilot implemented as intended?	Assess whether the policy reaches eligible participants; the processes involved in enrolment; whether take up and throughput match expectations; the extent to which the Welsh Government’s ‘4 conditions’ for the pilot are met (see: https://www.gov.wales/written-statement-basic-income-pilot-care-leavers-wales)	• Focus groups with managers (n = 6) • Focus groups with government officials (2) • Focus groups with Young Persons Advisors (YPAs) (n = 6) • Administrative data from Care Leaver teams/ the Welsh Government
Experiences: How is the pilot experienced?	Understand the experiences of participants and professionals involved; the role of the BIP in participants’ lives; transitions into and out of the pilot; attitudinal and behavioural changes	Interviews with participants/ supporter dyads (n = 40/ 20) Focus groups with PAs (n = 6) Focus groups with foster carers and housing support workers (n = 2)
Integration: How does the BIP fit into the overall offer for care leavers in Wales?	Consider how the BIP sits alongside other forms of support, whether its introduction changes this support (e.g. makes other things less attractive or accessible)	Focus groups with managers (n = 6) Focus groups with PAs (n = 6) • Focus group with financial advisors (n = 1) • Senior professional interviews (n = 7)

#### Data sources for IPE

The IPE will utilise data from two main sources: quantitative and qualitative monitoring administrative data gathered by the Welsh Government and qualitative data gathered directly from professionals and participants involved in the BIP, and their nominated supporters.

#### Recruitment to IPE

Participants of the IPE will be recruited through a range of means. Professionals will be invited via email. Young people who have consented for their contact details to be shared with the evaluation team will be invited by email or phone/ text. Invitations for supporters will be shared with eligible individuals by young people who nominate them, and they will contact the evaluation team if they are interested. Informed consent will be taken from all participants of interviews and focus groups.

#### Analysis within IPE

Monitoring data collected during the IPE will be analysed to provide descriptive and inferential statistics on the implementation of the pilot and characteristics of participants. Interview and focus group data will be subject to thematic analysis, a flexible method of identifying, analysing and interpreting patterns of meaning in qualitative data [70]. Braun and Clarke’s (2006) six-step approach to thematic analysis (familiarisation with the data; generating initial codes; searching for themes; reviewing themes; defining and naming them; producing the report) will provide systematic procedures for generating codes and developing themes. The analysis will be assisted by the use of NVivo software, which will aid management, consideration and visualisation of the data [71]. In order to generate and refine programme theory we will code qualitative data for key components of the intervention, contexts, mechanisms, and outcomes (CMO’s) in order to delineate how participants perceive the pilot to work. Transcripts from interviews and focus groups, fieldnotes from observations will be read and coded within the NVivo software package. Coded data will then be compared, contrasted and combined, before being represented visually in logic models and described in narrative form. These will be updated during the study as new data becomes available.

### Economic evaluation

The economic evaluation will take a public sector and a societal perspective to identify, measure and value the costs and outcomes of BIP, and synthesise the evidence to inform a social cost-benefit analysis. An additional cost-consequences analysis will present costs and consequences in a disaggregated form, together with the estimates of the mean costs of the comparator interventions with appropriate measures of dispersion. They are recommended for complex interventions that may have multiple implications [72], and for public health interventions which may have an array of benefits that are difficult to synthesise in a common unit such as cost-benefit [73]. The economic evaluation will be guided by a full economic analysis plan, and will be conducted alongside the impact evaluation, using the same research design and framework.

#### Data sources for economic data

Data will be collected from multiple sources and linked, using a mixed methods approach dependent on identified resource-use patterns and the availability of data. The YLBC survey, extended with extra questions for validated measures of well-being and economic impact, will provide baseline data. Throughput and associated costs data will be collected from the client, including use of the St David’s Day Fund and emergency grants. Data will be collected from the comparator cohort to identify baseline funding and other sources of economic engagement. LEO will provide secondary measures extending the cost-consequences analysis to educational and economic engagement, and to other health and social outcomes.

Data items will be captured in disaggregated units where possible, and micro-costing will be performed to capture variance in costing patterns. Unit costs for each resource input will largely be derived from national secondary sources, for example the Department of Health & Social Care’s NHS Reference Costs, the Personal Social Services Resource Unit (PSSRU), Office for National Statistics (ONS). They will be supplemented where necessary using primary research methods. The currency used will be expressed in British Pound Sterling (£), for a base cost year 2024/2025. Adjustments will be made for inflation using the PSSRU hospital & community health services index, and social service resource inputs index. All costs accrued beyond 12 months’ follow-up will be discounted to present values using nationally recommended discount rates [74,75].

#### Statistical methods for economic analysis

Specification of comparators and approaches for accounting for selection biases will mirror those planned for the impact evaluation [76,77]. Value for money will initially be expressed in terms of social cost-benefit at 24 months post-intervention, converting outcomes to monetary values. Accepted guidelines outlined in the HM Treasury Green Book [78] will be followed, constructed to explore the stated objectives of economy, efficiency and effectiveness.

The analysis will be informed by a comprehensive review of the broader literature regarding interventions similar to basic income, and appraisals of that funding approach. The full analysis model will include all cost and outcomes variables, in accordance with the “intention to treat” (ITT) principle. The cost-consequences analysis will make explicit the full range of the intervention’s impacts in disaggregate form. Costs and benefits will be estimated using subjective wellbeing evidence, which aims to capture the direct impact of a policy on wellbeing and broader social impacts such as engagement in education, financial literacy, and psychological well-being. Principles of opportunity cost will underpin all calculations. Missing data from either self-report, linked data or patient surveys will be imputed where appropriate to reduce the impact of missing data on regression results. A range of sensitivity analyses will be conducted to explore the impact of uncertainty surrounding key components of the economic evaluation on economic outcomes. These will be carried out for key costs and outcomes, specifically where they are highly sensitive to certain values or input variables. Sub-group analyses will mirror those undertaken for the main analysis. Summary statistics and cluster analysis may be used to determine data characteristics. Finally, narrative techniques will be used for outcomes which cannot be monetised, or where further exploration will be important, such as financial levers and incentives, mechanisms of change and unintended consequences.

### Project management

#### Data management plans

As data controller, the Welsh Government have collected data directly from participants with the support of the local authorities. Participants are made aware that the information they provide will be passed on to CASCADE with the option to opt out of this data sharing during enrolment. The evaluation team and Coram Voice are data processors. They receive and have direct access to survey data, as set out in Privacy Notices and information documents.

All data will be stored on Cardiff University servers in restricted folders available only to team members who require access. Data cleaning will be a regular process and data queries will be raised with the Welsh Government and Coram Voice if any discrepancies are found. All data queries will be logged within the tracker, and an audit trail maintained recording any changes to the data. Upon completion of data checks, the data manager will add the data to the master dataset and log the process as complete in the tracker. The following management plans are in place for each type of data.

***Survey data***. Survey data will be made available to the evaluation team by Coram Voice, via secure data transfer and through the evaluators having access to the ‘Smart survey’ software. Data will be checked, pseudonymised, and prepared for onward sharing to the impact evaluation team at KCL and the economics team at the University of Oxford, who will access databases via the Cardiff University secure server.

***Administrative data***. Administrative data from the LEO database will be accessed via applications to the Office for National Statistics (English LEO data) and the Welsh Government (Welsh LEO data), and processed within secure environments (e.g. the WISERD education data lab within the social science research park (SPARK) in Cardiff). Administrative data from monitoring forms will be made available to the evaluation team by WG via regular secure data transfers (using Objective Connect). It will be checked and stored on the Cardiff University secure server and deleted at the end of the study in accordance with funder terms and conditions.

Progress will be recorded in a tracking system and all submissions will be quality checked.

Strict data checks will also be completed upon receipt of data collection proformas. The data manager will conduct data cleaning at each time point to ensure there are no missing/duplicated data or any outliers.

### Confidentiality and data security

The management plans detailed above will ensure all data is stored securely and processed in accordance with data protection legislation (in accordance with GDPR and UK DPA 18) and Good Clinical Practice (GCP).

#### Ethical considerations

The study has ethical approval from the School of Social Sciences Research Ethics Committee of Cardiff University (Ref: SREC/323). Informed consent to participate will be obtained from all participants. Due to the use of remote methods, and in case consent forms are not returned, verbal consent will be audio recorded in interviews with professionals in addition to written consent.

If interviewees say anything that makes the researcher concerned about harm to the participant or another person, then they have a duty to take appropriate action. In the first instance, usually this would involve discussing the concern with the Principal Investigators or a co-investigator. Depending on the nature of the harm, referrals to agencies may be appropriate, for example a referral to the local authority children’s or adults social care services may be deemed necessary if someone was thought to be at risk.

#### Study status

The evaluation is underway, following an inception meeting with the Welsh Government on 23rd November 2022. The pilot began several months prior to this, on 1^st^ July 2022.

## Discussion

This study represents an unprecedented opportunity to understand the impact of a basic income scheme on care leavers, who are a particularly disadvantaged group. The uniqueness of the intervention means that the findings of this evaluation are likely to have a worldwide impact. The evidence it generates about support for care leavers, basic income schemes, and social security more generally is likely to be far reaching. We have designed the study with the limitations of previous basic income pilots in mind. We anticipate being able to provide robust estimates of impact on several key indicators and of value for money, and rich descriptions of the implementation and experiences of those involved.

Nevertheless, there are several challenges. Timing is a particular constraint, since the evaluation was commissioned four months into the 12-month enrolment period. This has two major implications. First, it affects the measurement of baseline outcomes using surveys. Self-report data gathered through surveys is optimal for many of the indicators that a basic income is theorized to affect, such as wellbeing, confidence and mental health indicators, so the Your Life Before Care survey is an important aspect of the evaluation. The survey was designed for (and in collaboration with) care leavers, has been used extensively with this population [6], and builds on previous work with looked after children [79]. Yet it was not designed for a basic income pilot, and the unamended survey used until January 2023 did not include some key questions about outcomes of interest. Thus, our baseline data is substantially determined by the content of a survey that was not designed with the evaluation of this pilot in mind.

The second implication of the timing of the evaluation relates to the amount of baseline data available from surveys. While the separate commissioning of Coram Voice to gather survey data at baseline was intended to ameliorate the delay in the evaluation starting, there were also delays in the survey being established, and problems with response rates. The first surveys were completed in October 2022 (3 months after the first participants enrolled), and response rates were unacceptably low (6% in November 2022). Problems with the way surveys were distributed were identified, and processes were consequently simplified and enhanced. This included a ‘thank you’ payment for participants completing the survey, and targeted communication with local authorities, and led to much higher response rates (35% by March 2023; 64% in September 2023 when the baseline intervention group survey was closed). This means some participants will have completed baseline surveys some time after the pilot started, and the data gathered therefore may not reflect the true baseline.

The wide range of outcomes of interest also creates a tension between breadth and brevity. In light of the initial low survey response rates, this has resulted in trade-offs between the extent to which we are able to include a wide range of validated measures and the need for surveys to be accessible and brief for those completing them.

Other challenges have caused us to amend aspects of the study design. We originally intended to run a contemporaneous survey with a matched comparator group of care leavers in England during the enrolment year period. We changed this plan in the first three months after being commissioned to conduct the evaluation. The timing of the start of the evaluation meant that, after allowing for the time period required to have access agreed with gatekeepers in England, the period where surveys in Wales and England could be completed contemporaneously was relatively small. The Welsh Government also favoured a within-Wales comparison for survey data. The change means comparisons for survey and administrative data will differ. We will compare care leavers in Wales in 2022-23 with care leavers in Wales in 2023-24 using survey data, and compare care leavers in Wales in 2022-23 with care leavers in England in 2022-23 using administrative data.

There are limitations and advantages associated with making this pragmatic change. The cohort of Welsh young adults who leave care between July 2023 and June 2024 will experience different labour market conditions due to both the effects of the COVID-19 pandemic and recent increases in the cost of living being experienced in the UK [80]. The latter in particular makes comparison on some indicators challenging. For example, levels of disposable income and bills may be markedly different for the later cohort if the trends of recent price rises continue. We will account for as much of this contextual variation as possible in in our analysis, but it will affect the confidence we can have in the findings. One advantage of the revised counterfactual plan is that comparator participants will be from the same (i.e. Welsh) local authorities rather than similar (i.e. matched English LAs). The use of triangulation between a range of different data sources, and of multiple analytical approaches, make the study somewhat resilient to these challenges.

Finally, it is important to note that the Welsh Government retain ownership and control of the data. They will publish the findings on their website (see below), and have published guidance that states “There must be no opportunity – or perception of opportunity – for the release of research information (unfavourable or not) to be altered, withheld or delayed for political reasons.” [81].

### Dissemination plans

We are contracted to report the findings in reports that will be published open access by the funder at https://www.gov.wales/statistics-and-research. We will also disseminate the study widely by other means, using in-person and online methods (e.g. conference presentations and invited talks), and by publishing in academic journals.

## Abbreviations

CSC: Children’s social care
EE: Economic evaluation
IPE: Implementation and process evaluation
DSA: Data sharing agreement
NPD: National pupil database
LA: Local authority
BIP: Basic Income for Care Leavers in Wales Pilot
YLBC: Your life beyond care survey
